# A LIVE ONLINE EXERCISE PROGRAM FOR OLDER ADULTS’ IMPROVED DEPRESSIVE SYMPTOMS: A PILOT RCT

**DOI:** 10.1093/geroni/igac059.979

**Published:** 2022-12-20

**Authors:** Giulia Coletta, Angelica McQuarrie, Julia Delvecchio, Paula Bochnak, Meridith Griffin, Ada Tang, Stuart Phillips

**Affiliations:** McMaster University, Hamilton, Ontario, Canada; McMaster University, Hamilton, Ontario, Canada; McMaster University, Hamilton, Ontario, Canada; McMaster University, Hamilton, Ontario, Canada; McMaster University, Hamilton, Ontario, Canada; McMaster University, Hamilton, Ontario, Canada; McMaster University, Hamilton, Ontario, Canada

## Abstract

Exercise improves mental health and effectively alleviates cognitive and physical declines. Unfortunately, engagement in physical activity decreases as individuals age and this was likely exacerbated by the COVID-19 pandemic. New technologies to deliver live online home-based group exercise classes may help mitigate mental and physical health declines in older adults while maintaining social connectivity. We evaluated the feasibility of an age-appropriate and ability-modified at-home exercise program via live video stream. The impact on loneliness, anxiety, and depression in older adults were exploratory outcomes. In this two-arm pilot RCT, we randomly assigned sedentary community-dwelling adults (65-80 years) to a waitlist control (CON) or an active group (ACTIVE) of thrice-weekly, 8-wk online live exercise program delivered via Zoom by trained exercise professionals. Attendance was recorded, and participant satisfaction to ACTIVE was assessed. Pre- and post-intervention loneliness, anxiety, and depression were collected using the revised UCLA Loneliness Scale (R-UCLA), the Geriatric Anxiety Inventory (GAI), and the Geriatric Depression Scale (GDS). 32 participants were randomized (ACTIVE: n=16, mean age 70 ± 4, 69% women, 30 ± 5 kg/m2; CON: n=16, mean age 71 ± 5; 88% women; 29 ± 5 kg/m2). Attendance to online classes was >80% and all ACTIVE participants reported being satisfied with the exercise sessions. There was no intervention effect compared to CON on loneliness and anxiety. An effect of the intervention was observed for depression (ACTIVE: -1.94; CON: -0.07; p=0.015). We demonstrated good feasibility, satisfaction, and preliminary efficacy of a live online exercise program on older adults’ mental health.

